# Tenascin-W: Discovery, Evolution, and Future Prospects

**DOI:** 10.3389/fimmu.2020.623305

**Published:** 2021-02-02

**Authors:** Martin Degen, Arnaud Scherberich, Richard P. Tucker

**Affiliations:** ^1^ Laboratory for Oral Molecular Biology, Department of Orthodontics and Dentofacial Orthopedics, University of Bern, Bern, Switzerland; ^2^ Tissue Engineering Laboratory, Department of Biomedicine, University Hospital Basel, University of Basel, Basel, Switzerland; ^3^ Department of Cell Biology and Human Anatomy, University of California at Davis, Davis, CA, United States

**Keywords:** extracellular matrix, cell adhesion, cell migration, cell differentiation, phylogeny

## Abstract

Of the four tenascins found in bony fish and tetrapods, tenascin-W is the least understood. It was first discovered in the zebrafish and later in mouse, where it was mistakenly named tenascin-N. Tenascin-W is expressed primarily in developing and mature bone, in a subset of stem cell niches, and in the stroma of many solid tumors. Phylogenetic studies show that it is the most recent tenascin to evolve, appearing first in bony fishes. Its expression in bone and the timing of its evolutionary appearance should direct future studies to its role in bone formation, in stem cell niches, and in the treatment and detection of cancer.

## Introduction

Tenascins are a family of extracellular matrix glycoproteins with a distinctive domain architecture (1). There are four family members: tenascin-C, tenascin-R, tenascin-X, and tenascin-W. All monomeric tenascins share a similar N-terminus comprised of heptad repeats flanked by cysteine residues. One or more epidermal growth factor (EGF)-like repeats are present near N-terminus, followed by variable numbers of fibronectin-type III (FNIII) domains. At the C-terminus is a large fibrinogen-related domain. Most tenascins form trimers *via* coiled-coil interactions within their N-terminal oligomerization domain, and some (tenascin-C and tenascin-W) can form hexamers through cysteine cross bridges. The original tenascin, and the best studied member of the family, is tenascin-C (1). The C in tenascin-C stands for cytotactin, which was one of the early names used for this protein. The second tenascin to be discovered was originally named restrictin since its expression is largely “restricted” to the nervous system. When it became clear that restrictin was closely related to tenascin-C, it was renamed tenascin-R. Tenascin-X received its name because it was encoded by the unknown “gene X”, which partly overlaps the human *CYP21B* gene. The domain architecture of tenascin-X is highly variable in different taxonomic groups, so its homolog in the chicken was initially named tenascin-Y until its true identity was discovered by phylogenetic analysis (2). The fourth and final member of the tenascin gene family is tenascin-W, which is the topic of this perspective article.

## The Discovery and Expression of Tenascin-W

In the decades prior to genomic sequencing, many researchers attempted to discover novel members of a gene family of interest by low-stringency hybridization screening of cDNA libraries with probes that corresponded to sequences likely to be conserved between members within the family. This approach was used by Philipp Weber and colleagues (3) to obtain the first tenascin-W sequence. In their study, a zebrafish cDNA library was screened for tenascin-related molecules using a radioactively labeled 444-bp sequence probe encoding the EGF repeats of rat tenascin-R. The 4 positive clones with sequences related to tenascins were amplified, subcloned and sequenced, and the missing 5’ sequence was determined by rapid amplification. While the experimental design seemed more likely to result in the cloning of tenascin-R from zebrafish, the resulting sequence appeared novel based on low sequence homologies with known tenascins. Weber and colleagues chose the name tenascin-W, presumably after the name of the discoverer. The cDNA sequence revealed a tenascin-type domain structure: an N-terminal signal peptide, a cysteine-rich domain likely to permit oligomerization, three EGF-like repeats, five FNIII domains, and a C-terminal fibrinogen-related domain.

The presence of a novel tenascin in zebrafish led to a race to find its mammalian homologue. The first of two papers describing the discovery of murine tenascin-W was published by John Neidhardt and colleagues (4). This group used BLAST to screen mouse expressed sequence tags (ESTs) for novel sequences encoding EGF repeats and FNIII domains, and found two overlapping sequences that did not correspond to the known sequences of murine tenascin-C and tenascin-R. The complete coding sequence was then found using rapid amplification and RT-PCR, revealing a tenascin with three EGF-like repeats, 12 FNIII domains and a C-terminal fibrinogen-related domain. Phylogenetic relationships between their novel tenascin and known tenascins placed the new murine tenascin in the tenascin-W clade. However, since it had 12 FNIII domains and not five as reported in zebrafish, they concluded that they had found a new tenascin and named it tenascin-N after the paper’s first author. The authors noted that many of the FNIII domains were very similar to each other, so much so that the amino acid sequences 6th and 8th FNIII domains were identical. They also reported that tenascin-N mRNA was abundant in brain, kidney and spleen, and less so in the developing embryo than in the adult mouse. The authors confirmed protein expression in the brain by immunohistochemistry.

The second paper describing tenascin-W in the mouse (5), submitted prior to the appearance of the paper by Neidhardt et al. (4), described the cloning of the homologue using PCR primers based on a human EST reported to be “similar to tenascin-R” (accession number AL049689). Overlapping cDNAs were assembled into a presumptive full-length coding sequence encoding 3 EGF-like repeats, 9 FNIII domains and a fibrinogen-related domain. The authors concluded that they found the murine homologue of tenascin-W as both the domain organization and sequence were most similar to zebrafish tenascin-W. They also noted that the similar FNIII domains (domains 3–8) corresponded to the 3rd and 4th FNIII domains of zebrafish tenascin-W, and they hypothesized that murine tenascin-W was larger than zebrafish tenascin-W due to the repetitive duplication of these domains over time. Immunohistochemistry revealed tenascin-W in developing smooth muscle and periosteum, in certain stem cell niches, as well as in the adult kidney. They did not report expression in the developing or adult central nervous system.

Why did the authors of these papers come to such different conclusions? We now know that the number of repeats or domains found in tenascins are very poor predictors of homology (1). Human tenascin-C has up to 17 FNIII domains, whereas its homologue in zebrafish (NP_570982) has nine. The human tenascin-X gene encodes 32 FNIII domains, whereas tenascin-X in some bony fish has only three. The numbers of EGF-like repeats are more similar across species, but even those repeats are highly variable in number in tenascin-X (2). The variable numbers of FNIII domains in tenascin-W (five in zebrafish and 12 in mouse) is likely to be the result of a duplication of the 3rd FNIII domain, which is encoded on a single exon (2). In the pufferfish tenascin-W has only four FNIII domains, but the 3rd domain duplicated twice in the zebrafish to give it six potential FNIII domains, one more than was sequenced by Weber et al. (3). Three copies of this domain are found in chicken tenascin-W, six in human, and nine in mouse. An exposed loop in this domain contains a potential integrin binding site, but this has yet to be demonstrated experimentally (2).

**Figure 1:**
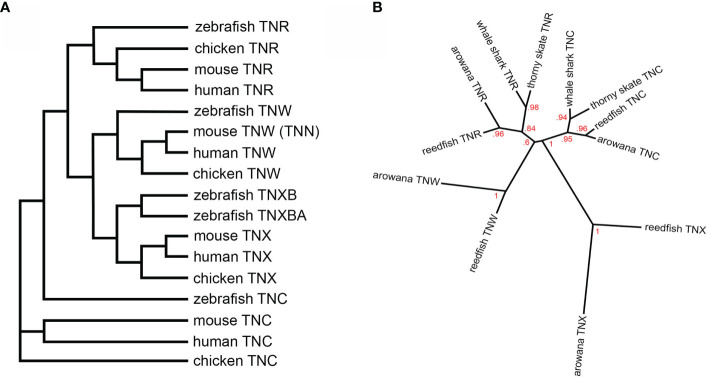
**(A)** A dendrogram constructed using the fibrinogen-related domain amino acid sequences of zebrafish, chicken, mouse and human tenascins. The tree was constructed using the default tools and parameters at NGPhylogeny.fr (5). The murine tenascin-W (TNW), originally named tenascin-N (TNN), segregates to the TNW clade. **(B)** A phylogenetic tree constructed using the fibrinogen-related domain amino-acid sequences of tenascin-C (TNC) and tenascin-R (TNR) from the cartilaginous thorny skate and whale shark, as well as the ancient bony reedfish and arowana, and from the fibrinogen-related domains of reedfish and arowana tenascin-X (TNX) and TNW. The tree was constructed using the same parameters as Adams et al. (6) and branch support is indicated. TNW co-evolved with the bony fishes, most likely from a duplication of the TNR gene. See text for details.

Phylogenetic tree construction is a more reliable way to identify homologues. When the fibrinogen-related domains of tenascins (the single large domain is easier to align than multiple—and variable—smaller domains) from zebrafish, chicken, mouse and human are used to establish their relationships using the phylogenetic tree-making program NGPhylogeny.fr (5), the mouse “tenascin-N” sequence clearly falls in the tenascin-W clade (**Figure 1A**). Similar results have been published by others (see below).

To date, the only evidence of tenascin-W expression in the central nervous system comes from Neidhardt et al. (4). Others found that the primary sites of expression are developing palate, bone and smooth muscle, certain stem cell niches, and in adult kidney, spleen, and periosteum (7–12). The absence of significant tenascin-W expression in brain parenchyme was confirmed by the tissue-based map of the human proteome published by Uhlén at el. (13).

Tenascin-W is also highly expressed in solid tumors (14–18). Details of tenascin-W expression in cancer can be found in a recent review (19).

How is tenascin-W expression regulated? Most of the work published to date on this subject has been done using C2C12 cells, or preosteoblast or osteo-chondroprogenitors, and these studies indicate that BMP-2 can upregulate tenascin-W expression, either directly or indirectly, through a p38-dependent signaling pathway (12, 14, 20). TNF-alpha can upregulate tenascin-W in mouse embryo fibroblasts (14), and Wnt3a, Wnt5a, and shear stress can upregulate tenascin-W expression in pre-osteoblastic MCT3T3-E1 cells (12). The regulation by Wnts is p38 dependent, but regulation by shear stress is JNK dependent. Tenascin-W expression accompanies osteoblastic differentiation in primary cultures (9), in Kusa-A1 cells (21) and in C2C12 cells (7), but not in MCT3T3-E1 cells. In MCT3T3-E1 cells, tenascin-W expression dramatically decreases during osteogenic differentiation (12), and addition of exogenous tenascin-W to MCT3T3-E1 cells can inhibit their differentiation as well (20).

## The Evolution of Tenascin-W

Tenascins are relatively modern additions to the extracellular matrix. Tenascins or tenascin-like proteins are limited to the Phylum Chordata (2, 6, 22, 23), suggesting that tenascins co-evolved with a dorsal hollow nerve cord and pharyngeal apparatus. In amphioxus and tunicates there are single tenascin genes, and phylogenetic trees constructed using the sequences of the fibrinogen-related domains do not place these tenascins from invertebrate chordates in any particular clade. Two tenascins are found in the lamprey, and in cartilaginous fish (the elephant shark *Callorhinchus milii*) there are three: tenascin-C, tenascin-R, and tenascin-X (6). Tenascin-W is present in all Euteleostomi (i.e., all chordates with bones). Since that report, the genomes of the thorny skate *Amblyraja radiata* and the whale shark *Rhincodon typus* have been reported. As with the elephant shark, these cartilaginous fish have tenascin-C (XM_033049212.1; XM_020527269.1) and tenascin-R (XM_033029137.1; XM_020515183.1), but they do not appear to have a tenascin-W.

Did tenascin-W evolve from tenascin-C or tenascin-R? Phylogenetic trees based on the amino acid sequences of the fibrinogen-related domains from bony chordates have been inconclusive (6). The addition of the skate and whale shark sequences to the analysis does suggest a closer, but still tenuous, relationship to tenascin-R of ancient Actinopterygii *Erpetoichthys calabaricus* (the reedfish) and the ancient teleost arowana *Scleropages formosus* (**Figure 1B**). The best evidence of the origin of tenascin-W is the observation that in all genomes examined tenascin-W is found adjacent to tenascin-R encoded in the opposite orientation (2). For example, in the zebrafish tenascin-W and tenascin-R are found adjacent to each other on chromosome 2, with tenascin-W on the negative strand and tenascin-R on the positive strand. In the mouse, these genes are adjacent to each other on chromosome 1, and once again tenascin-W is on the negative strand and tenascin-R is on the positive strand. This indicates that tenascin-W likely evolved from an isolated reverse tandem duplication event, and not from whole genome duplication. The latter probably resulted in the duplication of tenascin-X in the zebrafish, as tenascin-XB (XP_021323869.1) is on chromosome 19 and tenascin-XBA (XP_02132258.1) is on chromosome 16; chromosomes 19 and 16 in the zebrafish share a large number of paralogous genes (24). Interestingly, many of the genes that were duplicated in zebrafish by tandem duplication events have been linked to immune pathways (24).

## Future Directions

Unlike tenascin-C, we still know very little about the basic biology of tenascin-W. It is typically abundant in solid tumors and can affect cancer cell behavior (19), but the significance of these observations is still unknown. It is possible that tenascin-W plays a role similar to tenascin-C in the creation of an immune-suppressive environment in tumor stroma (25), though this hypothesis needs to be tested. The fibrinogen-related domain of tenascin-W, like that of tenascin-C, can bind and activate TLR4, which indicates tenascin-W may play a role in inflammation and inflammatory diseases (26). In addition, its prominent expression in the periosteum, a dense connective tissue around the bones containing progenitor cells that develop into osteoblasts, and at sites of osteogenesis, suggests a role for tenascin-W in bone repair and remodeling. The stimulating effect of tenascin-W on osteoblastic progenitors’ differentiation and migration (9) seems to confirm this hypothesis, but contrary observations have also been published (20). It is likely that future studies of the role of tenascin-W in bone will not only clarify the role for this extracellular matrix in normal bone development and remodeling, but also after bone injury and in diseases. Such a function of tenascin-W remains to be demonstrated in animal models of bone fracture repair and of osteoporosis. The highly specific expression of tenascin-W in other (non-osteogenic) stem cell niches, like in the corneal limbus or cuspid niche of the aortic valve, strongly suggests a role in tissue homeostasis and repair after injury, which still requires experimental confirmation.

The authors of this perspective would also like to encourage those who study tenascin-W to use that name instead of tenascin-N, or at least include both in their descriptions. It seems more appropriate to recognize the original discover of the protein in zebrafish than to use the name that was attributed, incorrectly, by one of the first to find this protein in mouse almost 5 years later.

## Data Availability Statement

The original contributions presented in the study are included in the article/supplementary material. Further inquiries can be directed to the corresponding author.

## Author Contributions

Each author (MD, AS, and RT) contributed to the conception, writing, and/or final editing of this paper. All authors contributed to the article and approved the submitted version.

## Conflict of Interest

The authors declare that the research was conducted in the absence of any commercial or financial relationships that could be construed as a potential conflict of interest.
